# Aging phenotypes in cultured normal human mammary epithelial cells are correlated with decreased telomerase activity independent of telomere length

**DOI:** 10.1186/2041-9414-4-4

**Published:** 2013-05-29

**Authors:** Klara Sputova, James C Garbe, Fanny A Pelissier, Eric Chang, Martha R Stampfer, Mark A LaBarge

**Affiliations:** 1 Life Science Division, Lawrence Berkeley National Laboratory, Berkeley, CA 94720, USA

**Keywords:** Aging, Human mammary epithelial cell, HMEC, Telomere, Telomerase

## Abstract

**Background:**

Shortening of telomeres, which are essential for maintenance of genomic integrity, is a mechanism commonly associated with the aging process. Here we ascertained whether changes in telomere lengths or telomerase activity correlated with age in normal human mammary epithelial cells (HMEC), or with phenotypes of aging in breast. Accordingly, flow cytometry fluorescence in situ hybridization (flowFISH) was used to determine relative telomere lengths (RTL), and telomerase activity was measured by the telomeric repeat amplification protocol (TRAP), in a collection of 41 primary HMEC strains established from women aged 16 to 91 years.

**Results:**

RTL measurements of HMEC strains that were heterogeneous with respect to lineage composition revealed no significant associations between telomere length with age, maximum observed population doublings, or with lineage composition of the strains. However, within strains, luminal epithelial and cKit-expressing epithelial progenitor cells that were flow cytometry-enriched from individual HMEC strains exhibited significantly shorter telomeres relative to isogenic myoepithelial cells (P < 0.01). In unsorted strains, detectable telomerase activity did not correlate with RTL. Telomerase activity declined with age; the average age of strains that exhibited TRAP activity was 29.7 ± 3.9y, whereas the average age of strains with no detectable TRAP activity was 49.0 ± 4.9y (P < 0.01). Non-detectable TRAP activity also was correlated with phenotypes of aging previously described in HMEC strains; increased proportions of CD227-expressing luminal epithelial cells (P < 0.05) and cKit-expressing progenitor cells (P < 0.05).

**Conclusions:**

Telomere shortening did not correlate with the chronological ages of HMEC strains, whereas decreased telomerase activity correlated with age and with lineage distribution phenotypes characteristic of aging.

## Background

The incidence of breast cancer increases dramatically with age. We have generated a large collection of cultured normal human mammary epithelial cells (HMEC) strains to better understand whether the aging process alters, the mammary epithelia in potentially deleterious ways [1]. This resource consists of multiple strains of normal finite lifespan pre-stasis HMEC established from discarded reduction mammoplasty and peripheral to tumour mastectomy tissues from women aged 16 to 91 years. Evaluation of lineage distributions within this diverse collection of pre-stasis HMEC strains revealed that, whereas myoepithelial (MEP) cells decreased proportionately, luminal epithelial (LEP) and cKit-expressing progenitors (cKit+) with a differentiation defect increased proportionately with age; comparison to uncultured samples and tissue specimens confirmed those changes also occur in vivo. Myoepithelial cells are thought to be tumour-suppressive [2,3], and progenitors are putative etiological roots of some mammary tumours [4-7]. The cell intrinsic changes that correlate with, or cause, these phenotypes of aging in HMEC are unknown. Because telomere shortening has been associated with aging and short stable telomeres are a characteristic of carcinomas we considered that age-dependent shortening of telomeres in HMEC could contribute to the observed age-associated changes.

Replication-dependent telomere shortening has been mechanistically linked to senescence in cultured fibroblasts [8]. Age-dependent, telomere shortening was reported in human lingual epithelium [9,10], kidney cortex [11], peripheral blood monocytes [12-14] and hematopoietic stem cells [15]. However, not all human tissues that have been examined exhibited age-dependent telomere shortening, for instance cerebral cortex and myocardium [16]; notably, these tissues are less proliferative in adults than blood and most epithelia. Age-dependent reduction in activity of the telomerase enzyme, which maintains telomere ends, also has been reported in a few normal human tissues, such as ovarian epithelia [17] and blood [18]. In a small number of normal cells adjacent to tumours in human breast tissue sections, telomere lengths measured with quantitative fluorescence in situ hybridization were found to vary by lineage such that telomeres of luminal epithelial cells were shorter than myoepithelial cells and stromal fibroblasts, but age-dependent shortening was not observed [19,20]. Generally, due to ease of access, blood has been the normal human tissue of choice for measuring age- and lineage-related telomere and telomerase changes, while there are fewer studies on solid human tissues. Due to the technical challenges of working in normal solid tissues, those studies often evaluated small numbers of cells and patients, and it has not been possible to associate functional phenotypes with changes in telomere or telomerase regulation. Our novel collection of normal primary HMEC strains, has enabled us to examine telomere lengths and telomerase activity to determine whether they were altered as functions of age or lineage, or correlated with the age-dependent increase of progenitors and LEP, or decreased MEP.

## Results

**Figure 1:**
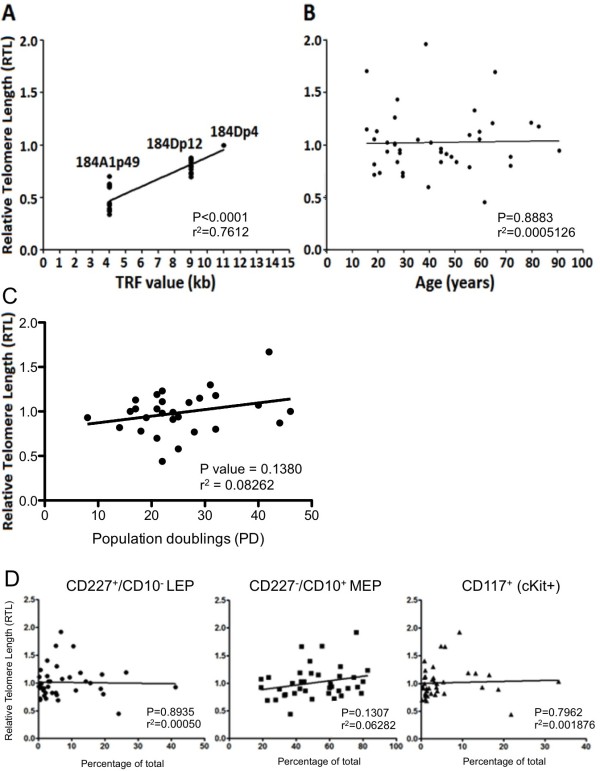
**No age**-**related telomere length reduction was observed in HMEC strains.** (**A**) A linear regression comparing Flow-FISH measured RTL values with mean TRF values in a well characterized pre-stasis strain and an immortal derivative with critically short telomeres, n = 6, r^2^ = 0.7612, P < 0.0001. (**B**) A linear regression of RTL measurements in 41 different passage 4 strains of normal pre-stasis HMEC as a function of age in years, r^2^ = 0.0005126, P = 0.8883. (**C**) A linear regression of RTL measurements in HMEC strains at passage 4 as a function total PD starting from passage 2, before the strains eventually entered stasis, n = 41, r^2^ = 0.08262, P = 0.1380. (**D**) Linear regressions comparing RTL measurements with proportions of (left) CD227^+^/CD10^-^ LEP (n = 41, r^2^ = 0.0005, P = 0.8935), (middle) CD227^-^/CD10^+^ MEP (n = 41, r^2^ = 0.0623, P = 0.1307), and (right) CD117/cKit^+^ HMEC (n = 41, r^2^ = 0.0018, P = 0.7962).

Relative telomere lengths (RTL) measured with flow cytometry-based fluorescence in situ hybridization (flow-FISH) were validated by comparing RTL to telomere lengths previously determined by mean telomere restriction fragment (TRF) Southern blots in a well-characterized normal HMEC strain and an immortal cell line derivative [21,22]. Normal pre-stasis finite lifespan (primary) 184D at passage 4 (~11.5 kb mean TRF) and passage 12 (~9.2 kb mean TRF) and immortal non-malignant 184A1 at passage 49 (~4.1 kb mean TRF) exhibited strong correlation with RTL values measured by flow-FISH that were normalized to 184D passage (p)4 (Figure 1A). Thus, flow-FISH RTL measurements were comparable to the TRF measurements obtained using Southern blotting, however RTL does not provide absolute length of the telomeres in question.

To determine whether telomere lengths differed as a function of age in HMEC, RTL were measured in 41 strains of pre-stasis finite lifespan HMEC at 4^th^ passage, whose donors ranged in age from 16 to 91 years. Linear regression analysis of RTL measurements showed no significant age-dependent changes in telomere lengths (Figure 1B), nor did RTL values show significant correlation with total population doublings that the HMEC strains underwent prior to arresting at stasis (Figure 1C). Previously, we reported age-dependent changes in the distributions of CD227+/CD10- LEP, CD227-/CD10+ MEP, and cKit + HMEC progenitors [1]. RTL values of the 41 strains at p4 also showed no correlation with the relative distribution of LEP, MEP, and cKit HMEC lineages (Figure 1D). Thus shortening of normal pre-stasis HMEC telomeres was not observed as functions of age or of lineage distributions.

**Figure 2:**
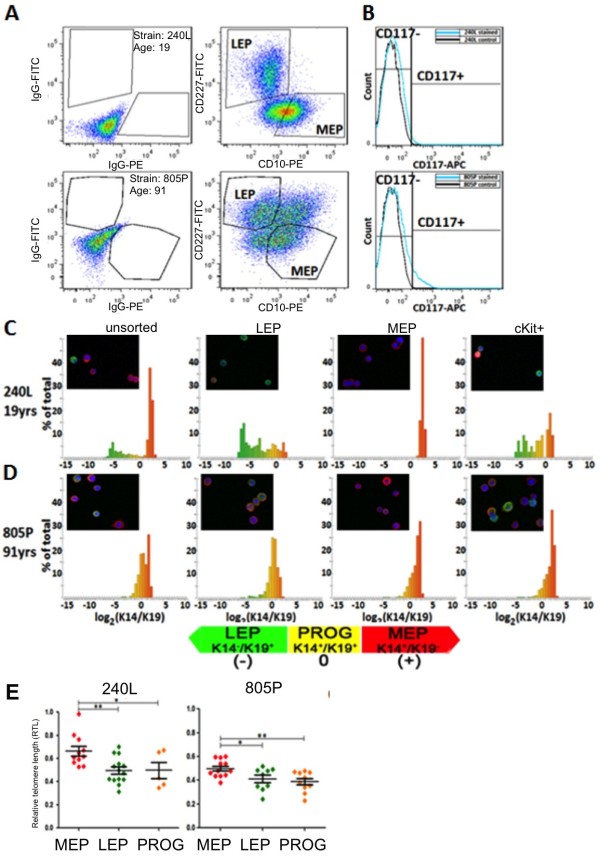
**Relative telomere length and telomerase activity varies by lineage within cultured strains.** (**A**) Representative FACS analyses of CD227 and CD10 expression in 4th passage HMEC strains isolated from a 19 year old woman (240L) and a 91 year old woman (805P). FACS plots are shown as dot plots, at left are isotype antibody controls, and at right are the CD10 and CD227 stained samples. Luminal and myoepithelial lineages are identified as LEP and MEP, respectively. (**B**) Histogram of CD117 expression by flow cytometry analysis of isotope negative control staining (black lines) and CD117 expression (blue lines) from 4th passage HMEC strains 240L and 805P. Histograms of ratios of keratin (K)14 to K19 protein expression in FACS enriched lineages, MEP (CD117-/CD227-/CD10+), LEP (CD117-/CD227+/CD10-), cKit + (CD117+) and unsorted cells from 4th passage strains (**C**) 240L and (**D**) 805P (n = 500 cells/histogram). Insets show examples of FACS enriched cells stained to show K14 (red), K19 (green), and nuclei (blue). (**E**) RTL measurements in FACS enriched lineages strains 240L and 805P at 4th passage, (n = 4). * = P < 0.05, ** = P < 0.01, ***P < 0.001.

To determine whether FACS-based RTL measurements would reveal that telomere length varied as a function of lineage in HMEC, as was previously reported from quantitative (Q)-FISH measurements in breast tissue sections [19,20], RTL were measured in LEP, MEP (Figure 2A), and cKit + lineages (Figure 2B) that were FACS-enriched from p4 HMEC strains derived from a 19y woman (strain 240L) and a 91y woman (strain 805P). Strain 805P had a larger proportion of CD227^+^ LEP and cKit^+^ progenitor HMEC compared to the younger 240L strain, as we predicted based on their relative ages. Enrichment of the three lineages was verified by automated image analysis of immunofluorescent staining for intermediate filament protein keratin (K)14 and K19 expression in sorted HMEC. Consistent with our previously reported observations of age-dependent lineage phenotypes, analysis confirmed that in HMEC from the 19y woman the MEP were enriched for the K14^+^/K19^-^ phenotype, LEP for the K14^-^/K19^+^ phenotype, and cKit progenitors for the K14^+^/K19^+^ phenotype (Figure 2C), whereas in the 91y woman all lineages were unusually enriched for K14 expression consistent with previous observations [1] (Figure 2D). Within both strains, the RTL significantly differed by lineage, where both LEP and cKit + cells exhibited shorter telomeres than the MEP (Figure 2E). Although significant, the differences in RTL between the lineages of the two strains were less than 1.5-fold, and the measurements of mixed populations, as in Figure 1B, essentially represent average RTL. If anything the average RTL of older strains would be biased in the direction of shortened telomere lengths, thus making our conclusion that HMEC telomeres are unlikely to shorten significantly with age all the more strongly.

**Figure 3:**
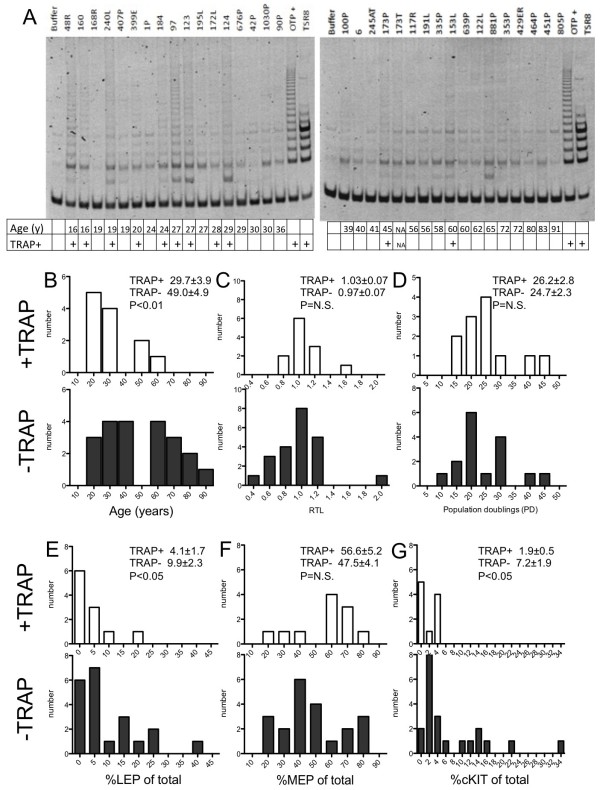
**Telomerase activity in HMEC decreases with age.** (**A**) TRAP gels for 34 strains of 4th passage pre-stasis HMEC. Histograms showing distributions, with their associated means, of (**B**) age, (**C**) RTL, (**D**) PD at stasis, (**E**) percentage LEP of total, (**F**) percentage MEP of total, and (**G**) percentage cKit + of total, in HMEC strains. Strains with TRAP activity are represented on the white histograms, and strains with no detectable activity are shown on the black histograms. Strain 173T, which are HMEC that originated from within a dissected breast tumour, was excluded from the analyses.

To determine whether telomerase activity changed with age, TRAP activity was measured in protein extracts from 34 HMEC strains at p4. Among the strains, 12 of 34 exhibited obvious ladders indicating TRAP activity (Figure 3A). The average age of HMEC strains that exhibited TRAP activity was 29.7y± 3.9y, whereas the average age of TRAP-negative strains was 49.0y± 4.9y (Figure 3B). This was confirmed by image quantification of the TRAP blots such that the ladder positive compared to the ladder negative group showed activity of 1.2 ± 0.1 versus 0.8 ± 0.1 relative arbitrary TRAP units (P = 0.03), respectively. TRAP activity did not correlate with RTL (Figure 3C) or with the number of population doublings the strains underwent before entering stasis (Figure 3D). Higher TRAP activity did correlate with decreased proportions of LEP (Figure 3E) and cKit + HMEC (Figure 3G) but did not correlate with proportions of MEP (Figure 3F). Telomere lengths and number of population doublings were independent of the level of detectable TRAP activity. However, more TRAP activity was detected in younger HMEC strains, and therefore also correlated with the age-dependent distributions of LEP and cKit^+^ progenitors. Consistent with suggestions that telomerase activity may be stress sensitive [23], we hypothesize that aging-related stresses lead to a global decline in telomerase activity within HMEC.

## Discussions

We have previously shown that cell intrinsic changes arise during the aging process, leading to an increase in proportions of LEPs and differentiation defective cKit^+^ multi potent progenitor HMEC, and decreased MEPs [1]. In this report, we are trying to understand what factors may contribute to those observations. Shortening telomeres have long been associated with tumour genesis and the aging process; however, not all tissues in humans that have been studied exhibited decreased telomere lengths with age. The tissue most often studied in the context of aging has been peripheral blood, due to its ease of access. We have used to our advantage the availability of breast tissues from cosmetic and mastectomy surgeries to study telomere changes in living normal human epithelial cells. In previous work in tissue sections of human mammary gland, the Q-FISH telomere-to-centromere length measuring method was used to assess telomeres in as many as 373 normal cells each from 30 different individuals, and the authors reported that telomeres in LEPs were shorter than in MEPs, but there were no signs of age-dependent shortening of telomeres across any lineage [19]. Here we report results of flow-FISH based RTL measurements on 10,000 normal pre-stasis finite lifespan HMEC each from reduction mammoplasty and peripheral to mastectomy tissues from 41 individuals, who spanned an age range from 16 to 91 years. RTL measurements revealed that telomeres in cultured LEPs and cKit^+^ progenitors were shorter than in isogenic MEPs. However, when viewed as whole unsorted strains, telomeres in HMEC did not exhibit shortening with age. The strength of that conclusion increases given that CD227-expressing LEP and cKit-expressing HMEC comprise a greater proportion of total cells (as much as ~50%, and ~25%, respectively) in the HMEC strains as age increases. In addition, telomere lengths in unsorted HMEC did not correlate with age-dependent lineage distributions, or the total number of population doublings that a strain underwent prior to entering stasis. The latter is consistent with a previous report that stasis in normal finite lifespan HMEC is a stress-related telomere-independent senescence barrier in which telomeres are not critically short [21]. Thus telomere shortening in cultured normal pre-stasis finite lifespan HMEC is unlikely to be a significant mechanism in driving the age-related phenotypes we previously reported, however, the sensitivity of the RTL assay may be less than what would be required to detect small differences.

Decreased telomerase activity has been reported with age in a few tissues [17,18], and different levels of telomerase activity have been observed in different hematopoietic lineages that are related by common progenitors [24]. In our study, TRAP activity was neither correlated with RTL nor with total number of population doublings (PD) that a strain underwent prior to stasis, but was associated with age, being lowest in older women. The lack of correlation between TRAP activity and PD was not unreasonable considering that HMEC stasis is achieved by a stress-related p16 mediated telomere-independent mechanism [21]. The reasons underlying decreased telomerase activity are important to consider and others have proposed that protracted age-related stress can cause decreased telomerase activity in humans [23]. The data presented here may represent the effects of aging-related stresses that caused an overall lowering of TRAP activity in older women’s HMEC. It is tempting to speculate that our observation of the age-associated emergence of more basal-like phenotypes in LEP, and the inability of older cKit+ progenitors to completely differentiate, may be related to these changes in telomerase activities.

Perhaps there may be a yet-to-be identified role for telomerase in the aging process that is distinct from maintenance of telomeres. For instance, telomerase expression has been shown to alter the regulation of the TGF-β, insulin, and EGF pathways, and vulnerability to oncogene-induced senescence, in HMEC [25-28]. Changes in IGF-related signalling and in IGF regulation are thought to partly explain age-dependent phenotypes in a number of tissues and model organisms, and changes in telomerase activity occur in response to IGF-1 signalling (reviewed in [29,30]). In addition, other non-telomere roles for telomerase have been reported to include anti-apoptosis, DNA repair, and transcriptional activation or repression that affects a large constellation of genes (reviewed in [31]). All of these possible non-telomere roles imply that gradual changes in telomerase activity over time, possibly due to aging-associated stresses, could have highly pleiotropic phenotypic consequences, and may contribute to age-dependent phenotypes in HMEC.

## Conclusions

Telomere shortening did not correlate with the chronological ages of HMEC strains, whereas decreased telomerase activity correlated with age and with lineage distribution phenotypes characteristic of aging. Strictly speaking, the shortening of telomeres may have little to do with healthy aging in the breast, but these findings leave open the possibility that decreases in telomerase activity may play a role in the aging process.

## Methods

### Cell culture

Cultured strains of finite-lifespan HMEC were established and maintained according to previously reported methods [32,33] and grown in M87A media with oxytocin and cholera toxin [21]. Tissues for establishing the strains were obtained as surgical discard material from local hospitals. Written consent to use tissue for biomedical research was obtained by the hospital prior to surgery from the patient or guardian. These cultures are de-identified. All work with human derived material was reviewed and approved by the Human Subjects Protection Committee at the Lawrence Berkeley National Laboratory.

### Immunofluorescence

Cells were cultured on glass cover slips until sub-confluent. Cells were fixed in methanol: acetone (1:1) at −20°C for 20 minutes, blocked with PBS/5% normal goat serum/0.1% Triton X-100, and incubated with anti-keratin (K)14 (1:1000, Covance, polyclonal) and anti-K19 (1:10, Developmental Studies Hybridoma Bank, clone Troma-III) overnight at 4°C, then visualized with fluorescent secondary antibodies (Invitrogen). Cells were imaged with an LSM-710 Confocal Microscope (Carl Zeiss). K14 and K19 expression levels were determined on a single cell basis using watershed-based cell segmentation algorithms (MatLab software by Mathworks).

### Flow cytometry

For identification of LEP, MEP, and cKit + progenitor lineages, anti-CD227-FITC (1:50, Becton Dickinson, clone HMPV) and anti-CD10-PE (1:25, BioLegend, clone HI10a), or anti-CD117-APC (1:50, BioLegend, clone 104D2), respectively were added to cells suspended in ice-cold M87A media for 25 minutes on ice. Cells were washed in PBS and resuspended in FACS buffer and sorted, using FACS Vantage flow cytometer with FACSDIVA software (Becton Dickinson).

### Telomere fluorescence in situ hybridization and flow cytometry (Flow FISH)

Telomere lengths of HMEC were measured with the DAKO Telomere PNA Kit/FITC for Flow Cytometry (DAKO) following the manufacturers protocol [34]. Briefly, relative telomere length (RTL) was determined by normalizing sample cells to 4^th^ passage (p) 184D, a cultured cell strain with previously well-defined telomere length and ploidy [21]. A total of 5 × 10^5 cells were resuspended in 150ul of hybridization solution containing 70% formamide with either no probe (unstained control) or with a fluorescein-conjugated telomere PNA probe. The cells were heated for 10 min at 82°C followed by hybridization overnight (for 19 hrs) at 25°C in the dark. Cells were washed with DAKO Wash Solution and heated for 10min at 40°C, then resuspended in 0.5 ml of FxCycle™ Far Red stain (Invitrogen) and incubated at 2-8°C for minimum of 30 min in the dark. Samples were analyzed by flow cytometry (FACS Calibur, Becton Dickinson) with a minimum of 50,000 events collected per sample, and data were analyzed with FlowJo software (Tree Star, Inc) RTL values were calculated as the difference between the signal of the stained and unstained control, normalized to RTL values of 184D p4.

### Telomeric repeat amplification protocol (TRAP)

Strains of HMEC at 4^th^ passage were trypsinized, washed once with PBS, pelleted by centrifugation, and frozen at −80°C. Frozen pellets were thawed and resuspended in CHAPS Lysis Buffer and incubated on ice for 30 min. Samples were centrifuged at 12,000 × g for 20 min at 4°C, and the protein concentration of the supernatant was determined by A280 using a Nanodrop spectrophotometer (Thermo). The telomerase activity in each sample was detected using the TRAPEZE Telomerase Detection Kit (S7700, Millipore), gels were imaged on a Storm 860 Molecular Imager (Molecular Dynamics). Signal intensities for the TRAP reactions were measured using ImageQuant software (G.E. Healthcare). Relative telomerase activity was calculated by subtracting the background signal in the no extract control from all samples and normalizing the resulting value to the signal intensity of specimen 184.

### Statistical analysis

Graph pad Prism 5.0 for PC was used for all statistical analysis. Standard linear regression was used. Grouped analyses were performed with Bonferonni’s test for multiple comparisons and Bartlett’s test for equal variance when distributions were Gaussian. Multimodal distributions were compared with Mann–Whitney tests. Significance was established when P < 0.05.

## Abbreviations

HMEC: Human mammary epithelial cells; LEP: Luminal epithelial cell; MEP: Myoepithelial cell; RTL: Relative telomere length; TRAP: Telomeric repeat amplification protocol; PD: Population doublings.

## Competing interests

The authors declare that they have no competing interests.

## Authors' contributions

KS, JCG, FAP, EC, MRS, and MAL carried out the cell culture, molecular, and FACS analyses. MRS was the originator of the HMEC Bank. KS and MAL participated in design of the experiments. KS and MAL performs statistical analysis of the data. MAL conceived of the study and drafted the manuscript with MRS. All authors read and approved the final manuscript. KS and JCG contributed equally to this work.
